# Spermidine and other functional phytochemicals in soybean seeds: Spatial distribution as visualized by mass spectrometry imaging

**DOI:** 10.1002/fsn3.1356

**Published:** 2019-12-19

**Authors:** Tatsuya Sagara, Dhaka Ram Bhandari, Bernhard Spengler, Johann Vollmann

**Affiliations:** ^1^ Department of Crop Sciences University of Natural Resources and Life Sciences Vienna (BOKU) Tulln an der Donau Austria; ^2^ Institute of Inorganic and Analytical Chemistry Justus Liebig University Giessen Giessen Germany

**Keywords:** Isoflavones, mass spectrometry imaging, polyamines, soybean, spermidine

## Abstract

Soybean seeds contain phytochemicals such as polyamines and isoflavones, which have been identified as functional components mediating health benefits in association with the consumption of soy foods. While a clear picture of the spatial distribution of these components within the seed is lacking, such information would be important to enhance or reduce their concentration in respective foods through processing. Thus, the objective of the present study was to visualize the most relevant components with respect to their distribution in soybean seeds. Mature soybean seeds were subject to atmospheric‐pressure scanning‐microprobe matrix‐assisted laser desorption/ionization (AP‐SMALDI) combined with a Fourier‐transform orbital trapping mass spectrometer to generate high‐resolution chemical images of phytochemical distribution. Based on seed cross sections, differential distributions of functional components were found between soybean cotyledon and germ (shoot, hypocotyl, root) regions. Spermidine and spermine were present in higher concentrations in the germ rather than in cotyledons with highest concentrations in root and shoot meristem tissues. Differential concentrations of spermidine and other components between the germ and cotyledon regions were confirmed by seed fractioning. In contrast to polyamines spermidine and spermine, the different types of daidzein, glycitein, and genistein isoflavones were all visualized in root parenchyma tissue exclusively. Overall, mass spectrometry imaging of soybean seeds revealed clear insights into the differential distribution of functional phytochemicals. Based on their distribution and depending on specific needs, spermidine and isoflavones can either be enriched or reduced during food processing by separating cotyledon and germ fractions.

## Graphical abstract

Spermidine is mediating various health effects through the induction of intracellular autophagy. In soybean, spermidine concentration is highest in the germ region of the embryo (shoot and root meristem tissues) as revealed by mass spectrometry imaging. Thus, separation of soybean germs during processing and food production can generate raw materials for the development of food supplements enriched in spermidine.

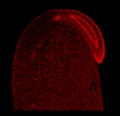

## 1 INTRODUCTION

The mature seed of soybean (*Glycine max* [L.] Merr.) is composed of about 40% proteins, 21% lipids, 34% carbohydrates, and 5% ash, based on dry matter. Due to the favorable nutritional composition, defatted soybean meal is widely used for livestock feeding. Additionally, soybean seed oil and processed and fermented soybeans are utilized in a large number of soy‐food products as well as in the food industry. Besides nutritional functions, a number of particular proteins, lipids, and minor soybean constituents such as saponins, isoflavones, vitamins, or nattokinases are beneficial to human health due to bioactive properties (Cao, Green‐Johnson, Buckley, & Lin, 2019; Medic, Atkinson, & Hurburgh, 2014). A further compound of interest is the polyamine spermidine, which is present in soybean seeds in significant amounts (Hou, He, Hu, & Wu, 2019; Sagara, Fiechter, Pachner, Mayer, & Vollmann, 2017). Spermidine has cardioprotective and neuroprotective effects mediated through the induction of autophagy (Madeo, Eisenberg, Pietrocola, & Kroemer, 2018), which might even restore immune functions in senescent lymphocytes (Zhang et al., 2019). Thus, spermidine‐rich foods probably are bearing additional health benefits to consumers. Soybean seed composition is variable, and the concentration of both major and minor constituents is differing among genotypes, geographic growing regions, and other environmental factors such as growing temperature, soil moisture, or agronomic practice (Medic et al., 2014). Within the seed, the distribution of individual constituents may be differing between cotyledons, embryonic axis, and the seed coat. For isoflavones and saponin precursors, the concentration is much higher in the embryo axis (commonly called germ) than in cotyledons and seed coat (Rupasinghe, Jackson, Poysa, & Kinjo, 2004; Tipkanon et al., 2011; Yue, Abdallah, & Xu, 2010), whereas the distribution of spermidine is not yet fully understood. Knowledge about the spatial distribution of different chemical compounds within a seed is important in food processing, as individual compounds of interest might either be enriched or reduced through fractioning of seed sections depending on specific needs in functional food development. In addition, the distribution of individual phytochemical components would also be relevant to highlight tissue‐specific processes in plant seed physiology research.

Analytical methods widely used in crop sciences such as near‐infrared reflectance spectroscopy (NIRS), high‐performance liquid chromatography (HPLC), and many others are based on detection of analytes from ground samples. In this case, the spatial information about analytes is lost. Recently, high‐resolution MS imaging methods based on matrix‐assisted laser desorption/ionization (MALDI) have been developed (Römpp & Spengler, 2013). These techniques combine information from mass spectrometry with the precise location of analytes, thus generating chemical images of individual analytes from MALDI matrix‐coated biological tissue sections. Besides animal and human tissue analysis, MS imaging has also been adapted to insect and plant tissues (Bhandari, Schott, Römpp, Vilcinskas, & Spengler, 2015; Bhandari, Wang, et al., 2015; Dong et al., 2016). Different plant organs such as root, stem, leaf, flower, fruit, and seed samples have been analyzed for various metabolites (Qin et al., 2018). Successful applications of MS imaging include licorice rhizome imaging of flavonoids or saponins (Li, Bhandari, Janfelt, Römpp, & Spengler, 2014), metabolites in maturing and germinating oilseed rape seeds, different tissues in wheat plants, and metabolites illustrating the infection of bread wheat seeds as well as stems with the *Fusarium graminearum* head blight disease (Bhandari, Wang, et al., 2015; Bhandari et al., 2018). In each of these cases, mass spectrometric images generated from various chemical compounds were in close correlation with optical images of tissue sections with different anatomical features, thus revealing the efficiency and wide applicability of the method.

So far, the distribution of polyamines and other functional phytochemicals within soybean seeds has not been described precisely. Therefore, the objective of this study was to adopt MS imaging for generating chemical images to map and examine the distribution of spermidine, isoflavones, and other compounds in mature soybean seeds. Detail knowledge about the location of these compounds would contribute to a better understanding of seed physiology processes as well as to the development of functional soy foods or dietary supplements with a high content of a particular compound.

## 2 MATERIALS AND METHODS

### 2.1 Plant materials

Individual seeds from soybean genotypes SOJA‐1902 and GP7X‐1871 were analyzed by MS imaging. SOJA‐1902 is an accession from the Gatersleben genebank (accession ID 93289; IPK Gatersleben) of soybean maturity group 000 with comparatively small seed size and high protein content. GP7X‐1871 (pedigree: GL601/ Vinton81) is a breeding line of maturity group 00 with larger seed size and lower protein content than SOJA‐1902. According to preceding analyses, seeds of SOJA‐1902 were significantly higher in concentrations of spermidine and the free amino acid arginine than GP7X‐1871. Seeds from both genotypes were harvested at full maturity from experimental field plots grown at Tulln an der Donau (Austria) during the 2016 season; air‐dried seed with a moisture content of 8% was stored at 5°C.

### 2.2 Preparation of samples for MALDI imaging

Sound soybean seeds were soaked on wetted filter paper for 24 hr. Subsequently, seeds were embedded in 4% (w/v) gelatin aqueous solution and frozen at −80°C for 60 min to form solid blocks. Thereafter, seed tissue sections of 20 µm thickness were obtained using a cryomicrotome (HM 525 cryostat, Thermo Scientific) operating at −20°C. Sections along the root–shoot axis and along one cotyledon were selected, thaw‐mounted on glass slides, and stored at −80°C prior to analysis.

Sections were brought to room temperature using a desiccator for avoiding condensation of humidity, and optical microscopic images of sections were captured before matrix application (Olympus BX‐41, Olympus Europa GmbH). For MS imaging of low molecular weight compounds in positive ion mode, a solution of 100 µl 2,5‐dihydroxybenzoic acid (30 mg/ml in acetone: water (50:50, v/v), 0.1% trifluoroacetic acid) was selected as a MALDI matrix and was sprayed onto the sections for coating using a pneumatic sprayer (SMALDIPrep, TransMIT GmbH).

### 2.3 Mass spectrometry and image generation

For generating mass spectra, an atmospheric‐pressure scanning‐microprobe matrix‐assisted laser desorption/ionization imaging source (AP‐SMALDI10, TransMIT GmbH) coupled to a Fourier‐transform orbital trapping mass spectrometer (Q Exactive, Thermo Fisher Scientific GmbH) was used. Soybean seed samples were analyzed in positive ion mode, and spectra were scanned at a mass‐to‐charge‐number (*m/z*) range of *m/z* = 80 to 800 at the mass resolution (R) of R = 140 000 at *m/z* of 200. Mass spectra for image generation were collected with 35 µm step size at an area of 8,750 × 7,700 µm (i.e., 250 × 220 pixels) and 8,575 × 8,400 µm (i.e., 245 × 240 pixels) for genotypes SOJA‐1902 and GP7X‐1871, respectively. Mass spectrometric images were generated using the software package MIRION, which creates ion images from selected *m/z* values within mass windows of ± 5 ppm (Bhandari, Wang, et al., 2015; Römpp & Spengler, 2013). Higher concentrations of a molecule are depicted in higher color intensity at the respective image position.

### 2.4 Seed fractioning and NIRS analysis

In two seed fractioning experiments for separating germ and cotyledon tissue, dry soybean seeds from two sets of different genotypes were individually cut into two parts close to the germ using a scalpel, and the respective seed parts were separated into fractions either with or without germ (see Table 1) for separate analysis of different compounds. Prior to near‐infrared reflectance spectroscopy (NIRS) analysis, seed parts (approx. 8–10 g per sample) were finely ground using a Cyclotec 1,093 mill (Foss Tecator). NIRS analysis for determination of seed protein, oil, sucrose, spermidine, and free arginine content was carried out using a Bruker Matrix‐I Fourier‐Transform NIRS instrument and OPUS software (Bruker) as described elsewhere (Sato, Zahlner, Berghofer, Lošák, & Vollmann, 2012; Vollmann, Walter, Sato, & Schweiger, 2011). All NIRS results were expressed on the basis of seed dry matter. The statistical significance of seed composition differences between the two corresponding fractions either with or without the germ section was determined using paired *t* tests.

**Table 1:** Compositional differences between soybean seed fractions with and without germ in two seed fractioning experiments and corresponding results of paired *t* tests

Trait	Seed fraction 1 (incl. germ)	Seed fraction 2 (without germ)	*t*‐value	*p* (2‐tailed)
Experiment 1 (20 soybean samples, approx. 25% fraction 1)				
Oil (g/kg)	160.6	167.2	2.7	.014
Protein (g/kg)	447.5	448.4	0.7	.515
Sucrose (g/kg)	61.0	61.8	1.0	.328
Spermidine (mg/kg)	229.7	224.1	2.1	.051
Arginine (mg/kg)	2086	1925	2.4	.028
Experiment 2 (6 soybean samples, approx. 22% fraction 1)				
Oil (g/kg)	148.7	194.6	12.9	<.001
Protein (g/kg)	440.4	422.0	4.7	.005
Sucrose (g/kg)	52.2	56.4	6.3	.001
Spermidine (mg/kg)	259.5	218.6	8.2	<.001
Arginine (mg/kg)	2,196	1,428	4.1	.009

## 3 RESULTS

### 3.1 Seed histology

**Figure 1:**
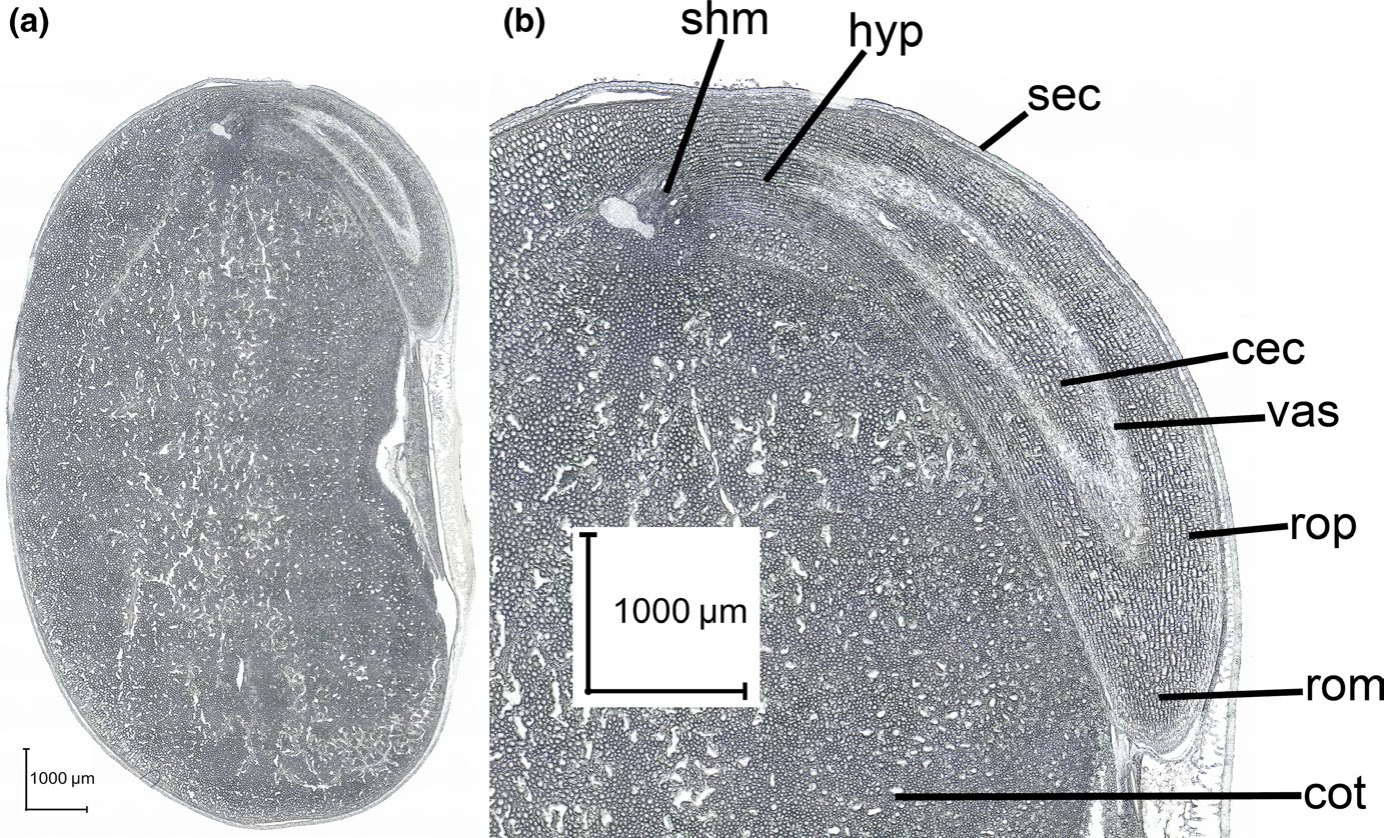
Optical image of the cross section of a mature soybean seed along the embryo axis and through a cotyledon (a); magnified germ region of seed (b) with shoot meristem (shm), hypocotyl region (hyp), seed coat (sec), central cylinder (cec), vascular system (vas), root parenchyma (rop), root meristem (rom), and cotyledon section (cot)

Cotyledons are making up the largest fraction of a soybean seed (Figure 1a, cross‐sectional overview), whereas germ and hilum regions represent much smaller structures. The magnified cross section from the germ division of the soybean seed (Figure 1b) reveals shoot and root meristem regions as well as a rather large radicle region with root parenchyma tissue, the vascular system, and the central cylinder clearly discernable.

### 3.2 Arginine, spermidine, and spermine distribution

**Figure 2:**
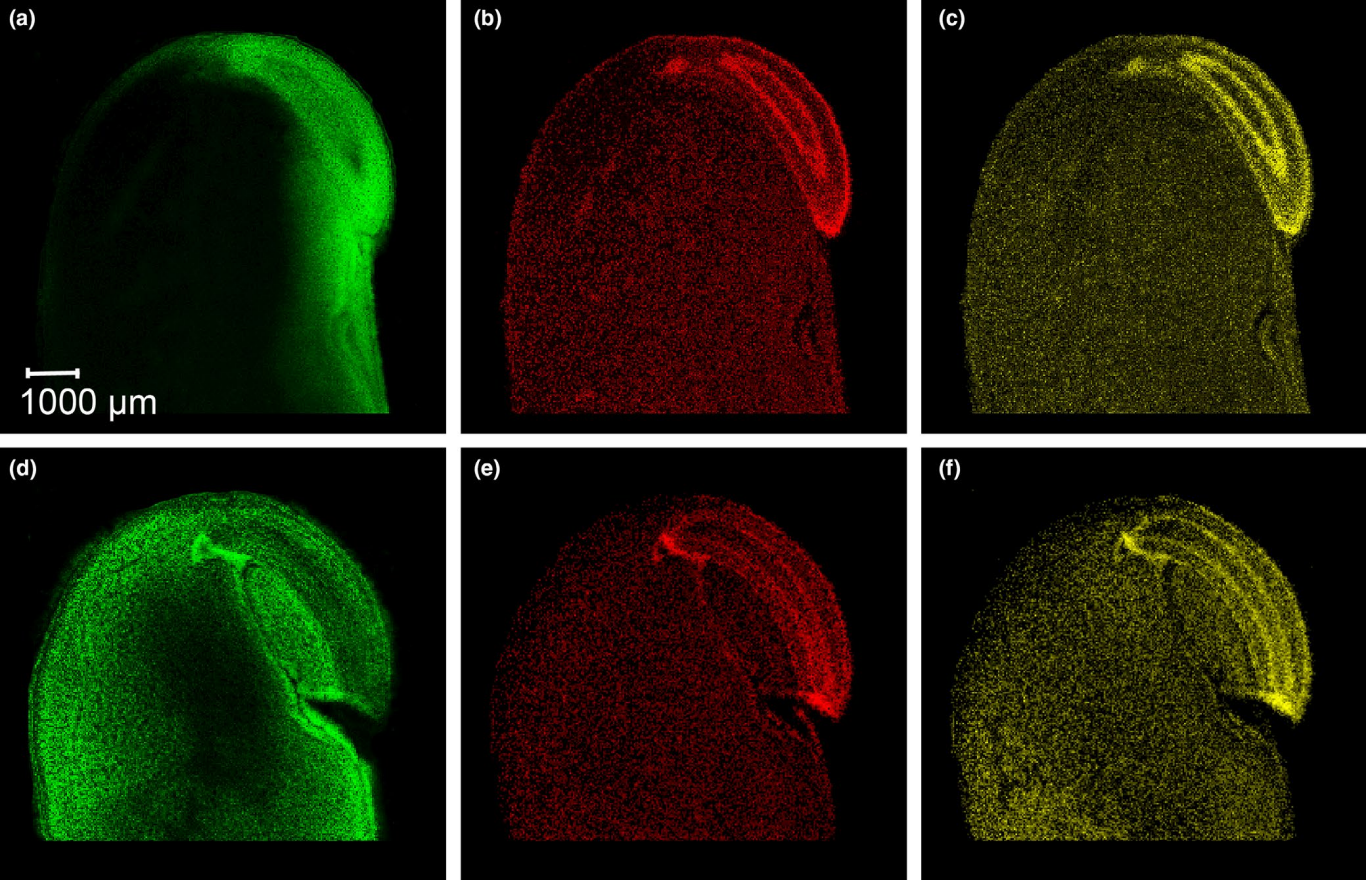
MS images revealing the distribution of arginine *m/z* 175.11895 [M + H]^+^ (a, d; green color), spermidine *m/z* 146.16517 [M + H]^+^ (b, e; red color), and spermine *m/z* 203.22302 [M + H]^+^ (c, f; yellow color) in cross sections of soybean seeds for genotypes GP7X‐1871 (upper row of pictures) and SOJA‐1902 (lower row), respectively

The free (non‐protein‐bound) amino acid arginine is visible in MS images both in germ and in cotyledon tissues (green color, Figure 2a,d). Genotype SOJA‐1902 (Figure 2d) was found to be higher in free arginine concentration than GP7X‐1871 (Figure 2a) based on MS imaging results, which is noticeable from higher green intensity in the cotyledon region in Figure 2d as compared to Figure 2a. The polyamines spermidine (red color, Figure 2b,e) and spermine (yellow color, Figure 2c,f) were found to share a similar distribution, and they were present at clearly higher concentrations in the germ compared to the cotyledon tissue in the investigated samples. Within the germ, regions of apical and root meristem, outer layers of root parenchyma and vascular system tissue exhibited higher concentrations of both spermidine and spermine as compared to their surrounding tissues in both genotypes. Genotype SOJA‐1902 had a higher overall spermidine concentration than genotype GP7X‐1871 in near‐infrared reflectance spectroscopy (NIRS) results, but this was not evident from MS images of the individual seeds analyzed here.

### 3.3 Isoflavones and other components

**Figure 3:**
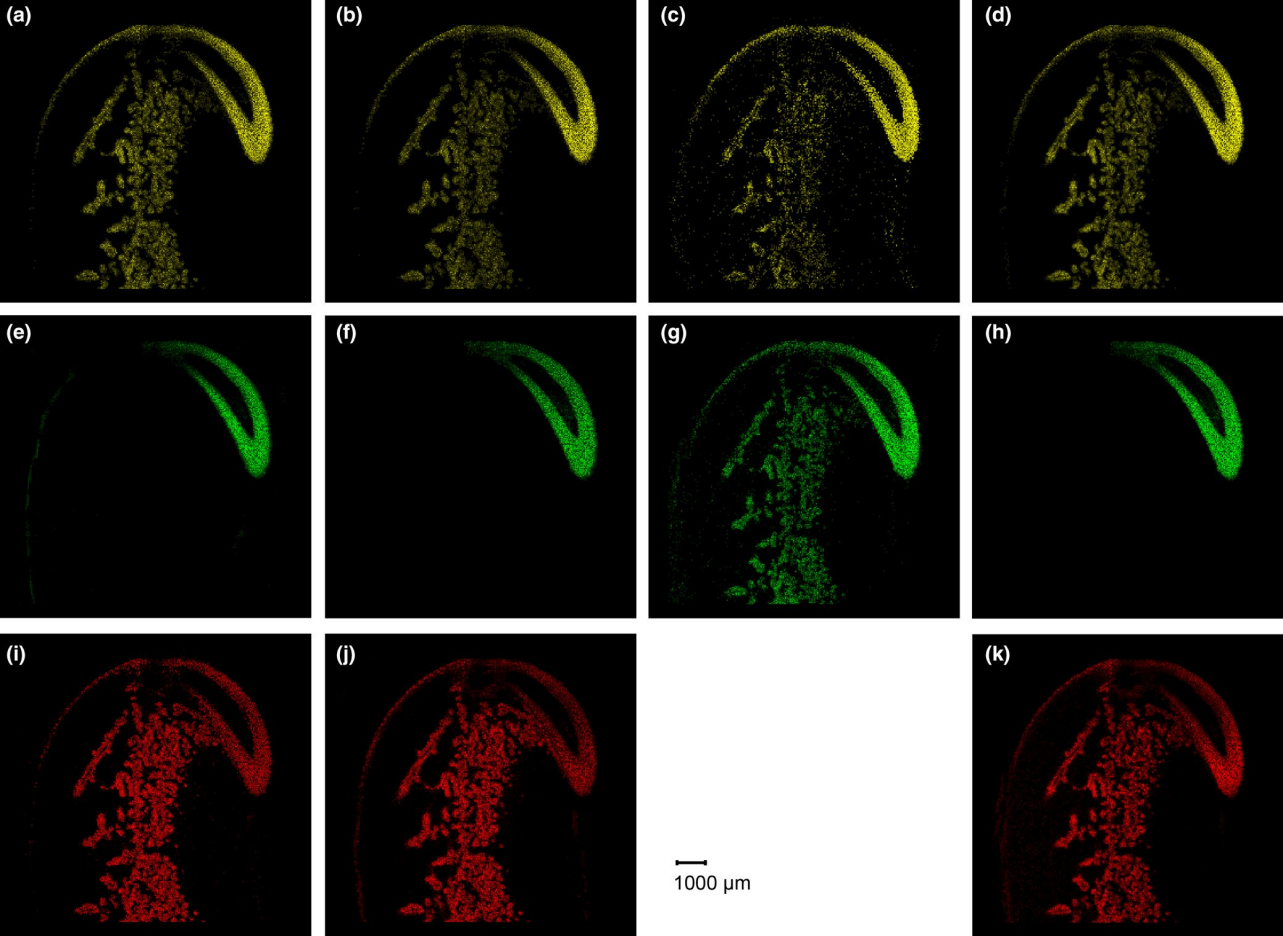
MS images of glycoside (a, e, i), malonyl (b, f, j), acetyl (c, g), and aglycone (d, h, k) types of the isoflavones daidzein (upper row), glycitein (middle row), and genistein (lower row), respectively, for genotype GP7X‐1871. Daidzin *m/z* 417.11856 [M + H]^+^ (a), glycitin *m/z* 447.12912 [M + H]^+^ (e), genistin *m/z* 433.11347 [M + H]^+^ (i), malonyldaidzin *m/z* 503.11895 [M + H]^+^ (b), malonylglycitin *m/z* 533.12952 [M + H]^+^ (f), malonylgenistin *m/z* 519.11387 [M + H]^+^ (j), acetyldaidzin *m/z* 497.08500 [M + K]^+^ (c), acetylglycitin *m/z* 527.09557 [M + K]^+^ (g), daidzein *m/z* 255.06519 [M + H]^+^ (d), glycitein *m/z* 285.07630 [M + H]^+^ (h), genistein *m/z* 271.0601 [M + H]^+^ (k)

Similar to polyamines, soybean isoflavones were present in high concentrations in germ tissue as well (Figure 3). In contrast to polyamines, isoflavones were found to be most enriched in root parenchyma tissues, whereas they were not present in the vascular system, the central cylinder, and the shoot section of the germ. In cotyledons, isoflavones were present at clearly lower concentrations as compared to root parenchyma tissue. The distributions of isoflavones were very similar for glycosides (i.e., daidzin, glycitin, genistin), malonyl isoflavones, acetyl isoflavones, and the aglycone forms (i.e., daidzein, glycitein, genistein).

**Figure 4:**
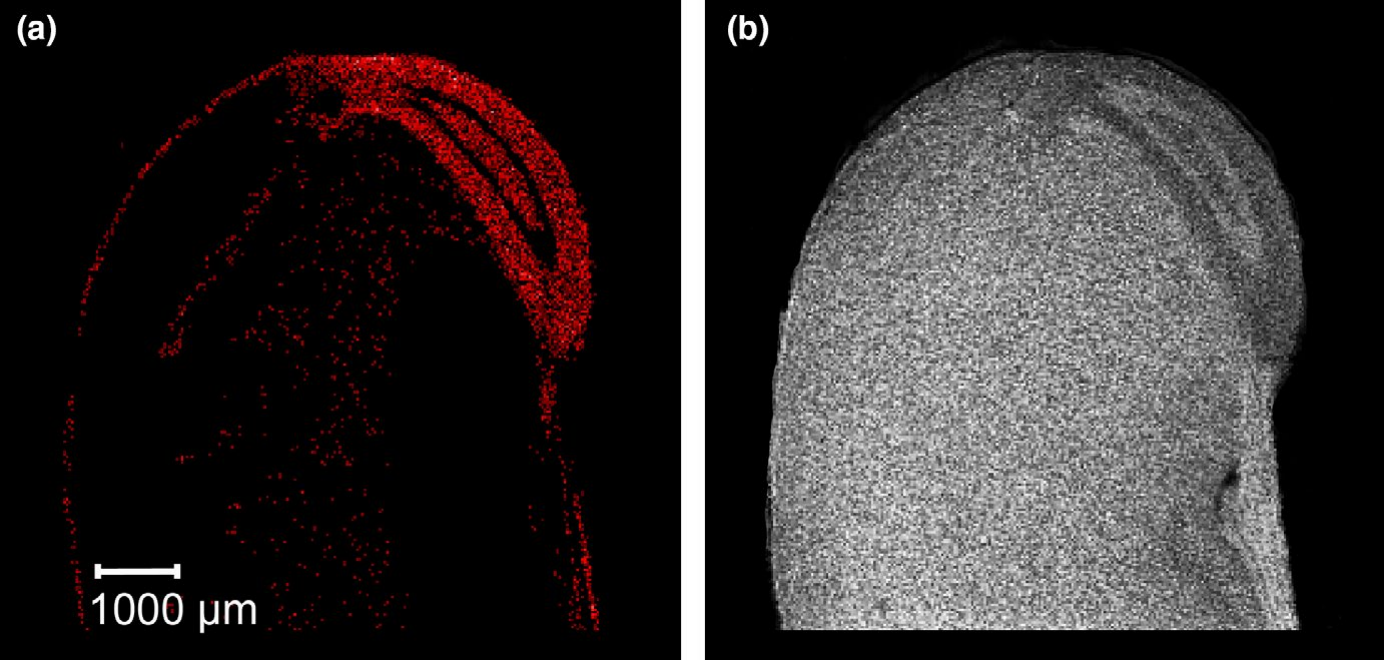
MS images of gamma‐tocopherol *m/z* 439.3552 [M + Na]^+^ (a) and sucrose *m/z* 365.10598 [M + Na]^+^ (b) in a seed section of soybean genotype GP7X‐1871

For other seed components, distributions were found to be different from those of polyamines or isoflavones. As an example, gamma‐tocopherol was present in most germ tissues except for the vascular system (Figure 4a). In contrast to gamma‐tocopherol, sucrose was mainly concentrated in the cotyledons, but was found in the vascular system of the germ section as well (Figure 4b).

### 3.4 Seed fractioning

As MS imaging results suggested differences in concentrations of individual compounds between soybean cotyledons and germ, seed fractioning experiments were carried out as a way of confirmation. In two different experiments with germ and cotyledon tissue separated, the fractions containing the germ were 25 and 22% of total seed mass, respectively (Table 1). In both experiments, the seed fraction containing the germ was found to be significantly higher in spermidine, arginine, and total seed protein content according to NIRS analysis results, whereas the fraction without germ was found higher in oil and sucrose content (Table 1). In experiment 2, seeds were larger than in experiment 1; thus a relatively smaller germ fraction (22%) could be cut off from the cotyledons causing a much higher concentration of arginine and spermidine in seed fraction 1 than in seed fraction 2 in experiment 2 as compared to experiment 1.

## 4 DISCUSSION

Differential distribution of several low molecular weight functional compounds of soybean seeds was visualized using MS imaging. This needs to be interpreted both in relation to the respective seed physiological roles of these compounds as well as to their impact on different soy foods and options for functional food development.

The localization of phytochemicals by MS imaging clearly differentiated compounds and seed's anatomical regions as connected with their respective biological function. The polyamines spermidine and spermine were found to be highly concentrated in meristematic tissues of the germ. However, they were present at lower concentrations in the cotyledons as well (Figure 2). The accumulation of spermidine in both apical and root meristem tissues indicates its major role in the regulation of gene expression (Miller‐Fleming, Olin‐Sandoval, Campbell, & Ralser, 2015). Moreover, polyamines have important functions in the protection from abiotic stress (Pál, Szalai, & Janda, 2015). A dormant soybean seed with low water content represents an osmotic stress to living and particularly meristematic cells. Therefore, the stress protection functions of spermidine and spermine might explain their accumulation in these tissues. Subsequently, in later stages of germination, spermidine and spermine concentrations were reduced in the radicle (Glória, Tavares‐Neto, Labanca, & Carvalho, 2005). In contrast, they remain rather constant in cotyledons, which appear as another indication of their osmotic stress protective function in meristematic tissue. The fact that both, elevated spermidine and spermine concentrations, are also present in the vascular system, indicates a connection between apical and root meristems with a rapid polyamine transport and signaling capacity. Furthermore, the high concentrations of spermidine and spermine in the root meristem regions are corroborated by the corresponding presence of the respective polyamine‐producing aminopropyltransferase enzymes, as previously demonstrated in transgenic *Arabidopsis* root models using immunohistochemistry and fluorescence confocal microscopy methods (Belda‐Palazón et al., 2012).

In contrast to polyamines, the main function of isoflavones is their signaling role in plant root–microbe interactions in the rhizosphere. Consequently, the different forms of isoflavones are all present in the root parenchyma tissue at high concentrations (Figure 3). This supports their rapid secretion through root exudates during plant development in order to initiate nodule formation and rhizobial symbiosis later on. In soybean, daidzein is secreted at highest rates during germination and early vegetative development stages for induction of nodulation (Sugiyama et al., 2016), whereas other isoflavones are secreted at later stages indicating a function in regulation of the soybean rhizosphere microbial communities through root exudates. Moreover, the present high‐resolution MS imaging results clearly reveal that the isoflavones are mainly accumulated in root parenchyma tissues of the radicle section of the germ, whereas in earlier investigations isoflavones were less precisely attributed to hypocotyl or embryo axis/germ regions of the seed (Berger, Rasolohery, Cazalis, & Daydé, 2008; Yue et al., 2010).

About 52%–60% of tocopherols in soybean oil are made up by gamma‐tocopherol (McCord et al., 2004). Although most of soybean lipids are stored in the cotyledons, gamma‐tocopherol is highly concentrated in germ tissues except for the vascular system (Figure 4a). A major biological function of tocopherols is protecting lipids from oxidation during seed storage and germination which is an essential component of seed longevity (Carrera & Seguin, 2016). Thus, the high concentration of gamma‐tocopherol found in the germ region of the seed appears to be due to that role of protecting seed viability. In cereals, a high tocopherol concentration is known from wheat germ as well (Brandolini & Hidalgo, 2012). Moreover, a very distinct distribution of alpha‐tocopherol has also been found in MS images of rice seeds, in which the highest concentration of tocopherol is present in a particular germ tissue, that is, the scutellum, only (Zaima, Goto‐Inoue, Hayasaka, & Setou, 2010).

Arginine is an important biosynthesis precursor of spermidine and spermine, and there is a positive correlation between arginine and spermidine concentrations. Differences in arginine concentration between a low (Figure 2a) and a high (Figure 2d) arginine soybean genotype can be recognized from the respective MS images. The low arginine genotype is also lower in spermidine, and arginine is mostly present in the germ rather than the cotyledon in that genotype (see also Table 1). A high content of free arginine particularly in the cotyledons might also indicate a deficiency mutation of a major storage protein fraction, by which free arginine has been reported to accumulate at higher levels as a storage reservoir of surplus nitrogen (Takahashi et al., 2003).

Beside the close connection between distribution of particular compounds and their respective physiological roles, the present results strongly support soybean processing toward the development of specialty soy‐food products and natural nutritional supplements with enhanced functional or health‐promoting compounds through seed fractioning. During seed maturation, soybean isoflavones accumulate in the germ section at much higher rates than in cotyledon and seed coat tissues (Berger et al., 2008; Yue et al., 2010). Thus, soybean germ flours are available with enriched isoflavone concentration which can be utilized in nutraceuticals or in the production of isoflavone extracts for medicinal applications (Imhof, Gocan, Imhof, & Schmidt, 2018; Tipkanon et al., 2011). As spermidine is enriched in the germ tissue similar to isoflavones (Figure 2b,e), seed fractioning (Table 1) could as well produce flours higher in spermidine for nutraceutical or food supplement uses. This would also apply to arginine (see Table 1, experiment 2). Moreover, the uneven distribution of spermidine between germ and cotyledons also serves as an explanation for the large variation in spermidine content found between different soy‐food products (Kalač, 2014; Toro‐Funes, Bosch‐Fuste, Latorre‐Moratalla, Veciana‐Nogués, & Vidal‐Carou, 2015). For instance, in the production of soy drinks, tofu, or protein isolates, most processors use cotyledons only while discarding germ and seed coat structures, which explains the low spermidine content found in most types of tofu, soy drinks, and similar products. This separation is carried out to avoid bitter or astringent off‐flavors in soy foods caused by certain isoflavones and saponins (Aldin, Reitmeier, & Murphy, 2006). In contrast, natto is well‐known as the soy food with highest spermidine content (Kalač, 2014; Kobayashi et al., 2017); for natto production, whole soybeans including the germ are utilized, and the spermidine content and other bioactive compounds are additionally enhanced in the final product due to microbial fermentation (Cao et al., 2019; Kobayashi et al., 2017). Moreover, the germ makes only 3%–4% of the total soybean seed mass, and the percent of germ tends to be higher in cultivars with smaller seed size (Tipkanon et al., 2011). This might as well support the particularly rich spermidine content of natto, as most types of natto are made of soybeans with small seed size and subsequently a larger germ percentage. Similar to natto, the Japanese food okara is high in polyamines spermidine and spermine (Takagi et al., 2009); and this is due to the fact that okara is a by‐product of soy milk and tofu production consisting of solid cotyledon fractions mainly, from which polyamines cannot be extracted during processing.

While presenting important options for functional soy food or nutrition supplement development, the present study is limited by a low number of seeds analyzed, which is due to the still costly and time‐consuming high‐resolution chemical imaging method applied. Subsequently, differences existing between soybean genotypes could not be addressed in terms of analyte concentration and distribution. Differences in analyte concentration between genotypes, analyte variation between individual seeds, and seasonal variation all need to be considered in future investigations, as they might affect the final concentration of functional food components.

## 5 CONCLUSION

High‐resolution mass spectrometry images of soybean seeds provide meaningful insights into soybean metabolomic and seed physiological processes such as the protection of meristematic cells during seed maturation and the germ/root location of isoflavones for rhizosphere signaling. With respect to food production, the results can support the development of functional soy foods or nutrition supplements based on seed fractioning and enrichment of functional chemical compounds such as polyamines, isoflavones, or others.

## CONFLICT OF INTEREST

B.S. is a consultant and D.R.B. is an employee of TransMIT GmbH. The other authors have no conflict of interest to declare.

## ETHICAL APPROVAL

This study did not involve any human or animal testing.
